# Gradenigo's Syndrome and Bacterial Meningitis in a Patient with a Petrous Apex Cholesterol Granuloma

**DOI:** 10.1155/2020/8822053

**Published:** 2020-10-20

**Authors:** Jacqueline Hodges, Julie Matsumoto, Nicholas Jaeger, Brian Wispelwey

**Affiliations:** ^1^Division of Infectious Diseases and International Health, University of Virginia, Charlottesville, USA; ^2^Department of Radiology and Medical Imaging, University of Virginia, Charlottesville, USA; ^3^Department of Pathology, University of Virginia, Charlottesville, USA

## Abstract

Gradenigo's syndrome (GS) classically involves a triad of ear pain due to acute or chronic otitis media (OM), facial or retro-orbital pain in the distribution of the trigeminal nerve, and an abducens nerve palsy. The simultaneous presentation of all three components has become less common in cases of GS reported in the literature, particularly in the era of antibiotics effective against typical organisms attributed to OM and petrous apicitis. In addition to infectious petrous apicitis arising directly from OM, more recent cases of GS are attributed to the compression of the same traversing cranial nerves in the presence of various expansile petrous apex (PA) lesions, both benign and malignant. We report a case of a 24-year-old male who presented initially with nausea, fever, photophobia, left-sided retro-orbital pain, and headache. He was diagnosed with bacterial meningitis by lumbar puncture and treated with empiric antibiotics, with CSF eventually revealing nontypeable *Haemophilus influenzae*. Several days into his course, he developed diplopia with leftward gaze. Brain imaging revealed an expansile, erosive PA cholesterol granuloma with associated contiguous dural and leptomeningeal enhancement. The patient improved with antibiotics and eventually underwent surgical intervention. This atypical presentation of GS with a rare complication of meningitis in the setting of a PA granuloma demonstrates the importance of early recognition of this syndrome, as well as consideration of added surgical intervention in patients with pre-existing petrous lesions at potentially higher risk of dangerous complications of GS.

## 1. Introduction

Gradenigo's syndrome (GS), first described in the literature by Giuseppe Gradenigo in 1907 [1], classically involves the triad of an abducens nerve (VI) palsy, facial pain in the trigeminal nerve (V) distribution, and suppurative otitis media (OM) with ear pain and otorrhea [1, 2]. The underlying pathophysiology involves introduction of bacterial organisms from middle ear infection to the mastoid air cells and medially into a pneumatized petrous apex (PA) [3]. Several important anatomic structures traverse the PA, including Dorello canal near the medial superior tip of the PA through which the abducens nerve passes, and the cisternal trigeminal nerve which crosses over the tip of the PA to enter Meckel cave [3].

Simultaneous clinical presentation with all three components of the triad, however, is less common in the literature [4], particularly in the era of widespread vaccination and use of antibiotics effective against typical organisms causing acute or chronic OM and subsequent petrous apicitis. Some reported cases of GS lack the triad's otic symptoms and are described as incomplete or nonclassical presentations [5–9]. There are multiple noninfectious lesions that may affect the petrous bone as well, including cholesterol granuloma, congenital and acquired cholesteatoma; benign and malignant osseous, chondroid, or dural-based lesions; and internal carotid artery (ICA) aneurysms [3, 10]. An increasing number of case presentations that remain labeled as GS or ‘mimics' of GS (due to cranial nerve V and VI involvement) are attributed to various petrous lesions [11–15] and lack preceding OM and mastoiditis.

In the majority of cases of bacterial meningitis, pathogenesis relies on an array of virulence factors that allow for mucosal attachment and survival within the bloodstream, followed by invasion through the blood-brain barrier [16]. Bacterial meningitis arising as a complication of GS, through direct introduction of bacteria into the cerebrospinal fluid (CSF) resulting from temporal bone erosion, has been reported sparingly in the literature [17–20]. We describe here a unique case of *Haemophilus influenzae* meningitis in an adult presenting with a nonclassical Gradenigo's syndrome and a previously undiagnosed petrous apex cholesterol granuloma. While an expansile petrous apex cholesterol granuloma itself has been attributed to presentation with GS [21], we found no cases in the literature describing simultaneous development of bacterial meningitis in a patient with GS and a pre-existing petrous cholesterol granuloma.

## 2. Case Presentation

A 24-year-old male with a history of well-controlled asthma presented to an outside hospital with 3 days of headaches he described as deep, worst behind the left eye, and radiating posteriorly, with associated fevers, nausea and vomiting, photophobia, and phonophobia. He denied hearing loss, tinnitus, ear pain, or ear drainage. Physical exam was without concern for active ear infection. Lumbar puncture was performed and revealed a neutrophil-predominant leukocytosis with a white blood cell count of 1759 cells/mm^3^. He was initiated on ceftriaxone and vancomycin empirically, and CSF culture and PCR revealed *Haemophilus influenzae* (later revealed to be a nontypeable strain). Six days into his hospital course, he developed a horizontal diplopia and discomfort upon attempted left eye abduction, concerning for a left abducens nerve (CN VI) palsy.

**Figure 1:**
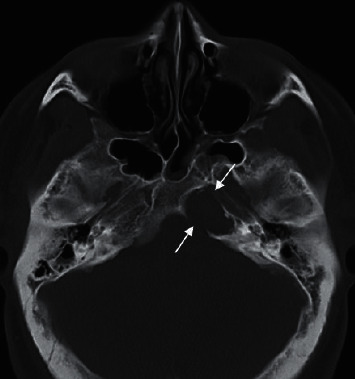
CT scan: left petrous apex smooth expansile lesion with internal soft tissue density and bone erosion.

**Figure 2:**
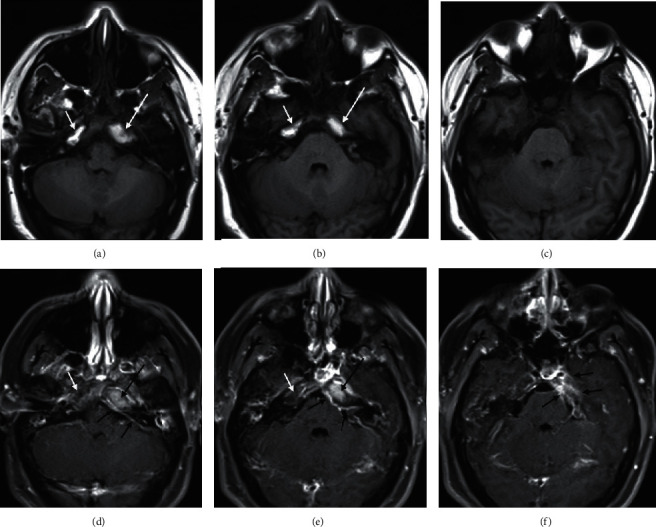
Axial T1 noncontrast (a–c), and postcontrast with fat-saturation (d–f): increased T1 signal in the left petrous apex lesion (a-b; long white arrows) that does not suppress on fat-saturated images (d-e; long black arrows), consistent with cholesterol granuloma. However, the thick rim of peripheral enhancement and abnormal meningeal enhancement (d–f, short black arrows) is consistent with a superimposed inflammatory process. Note that the right petrous apex also displays increased T1 signal (a-b; short white arrows), but becomes dark (“suppresses”) on fat-saturated images (d-e; short white arrows), consistent with normal fatty marrow.

**Figure 3:**
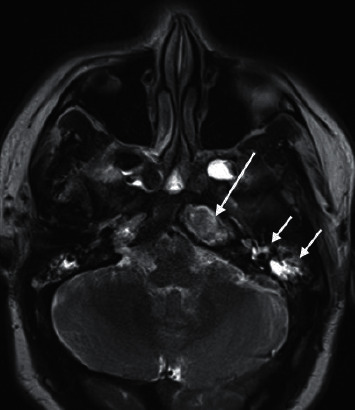
Axial T2: increased T2 signal in the left petrous apex lesion, typical for cholesterol granuloma (long arrow). Fluid signal in the left middle ear, mastoid air cells, and sphenoid sinus (short arrows).

**Figure 4:**
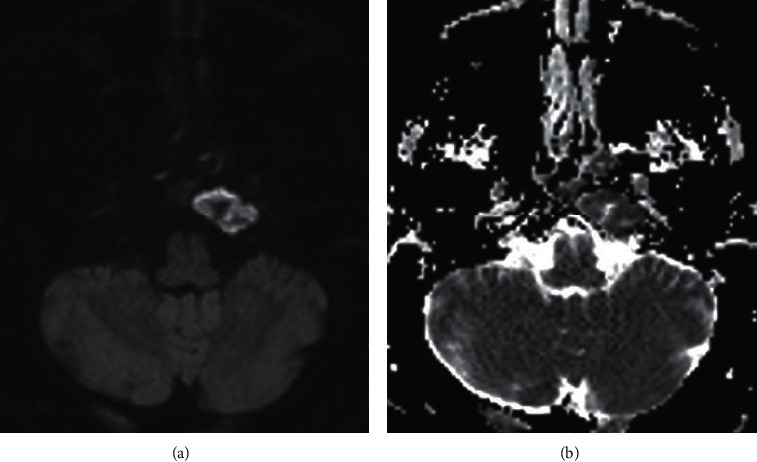
(a) Axial diffusion-weighted image (DWI). (b) Apparent diffusion coefficient image (ADC): heterogeneously restricted diffusion (increased signal on DWI and decreased signal on ADC) in the petrous apex lesion, likely reflecting viscous fluid. Simple cholesterol granulomas typically show decreased DWI signal.

**Figure 5:**
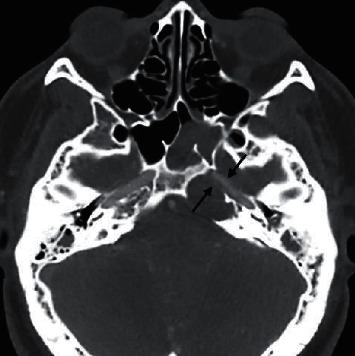
CT angiogram: the left carotid canal wall is dehiscent, and the ICA is narrowed (arrows), presumably due to vasospasm from adjacent inflammation. CTA is valuable to confirm that an expansile petrous apex lesion is not an ICA aneurysm.

CT scan of the head was obtained and revealed a 2.6 cm smoothly expansile left PA lesion with large areas of bone dehiscence around the margins (Figure 1). MRI demonstrated increased signal intensity within the lesion on noncontrast T1 images that did not suppress on fat-saturation images (Figure 2) and hyperintense signal on T2-weighted images (Figure 3), all of which are characteristic of cholesterol granulomas. However, atypical thick contrast enhancement around the periphery of the lesion and heterogeneous diffusion restriction suggested superimposed infection (Figures 2 and 4). Dural enhancement extended along the surfaces of the petrous temporal bone, internal auditory canal, and left tentorium. Leptomeningeal enhancement was present over the left pons, in the location of the cisternal CN VI, Dorello canal, and into Meckel cave (Figure 2). The left tympanic membrane appeared retracted, and there was enhancing left middle ear and bilateral mastoid fluid also visible. CTA confirmed smooth erosion of and displacement of the left carotid canal, and petrous ICA was narrowed, presumed secondary to vasospasm (Figure 5).

**Figure 6:**
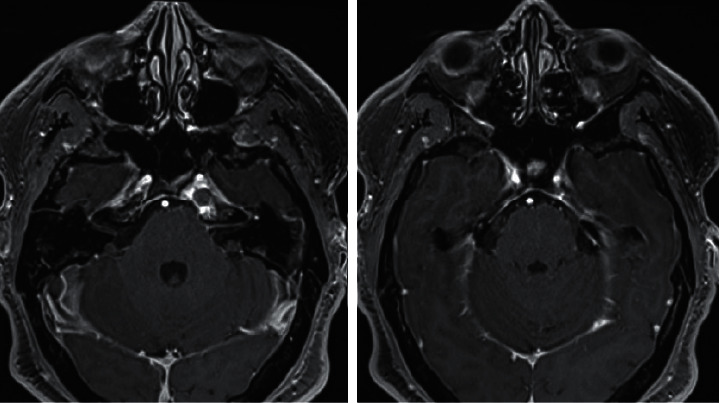
Axial T1 postcontrast with fat-saturation midway through antibiotic therapy: Leptomeningeal enhancement has resolved, and dural enhancement has decreased (compared to Figures 2(e) and 2(f)).

Following consultation with otolaryngology, ophthalmology, and neurosurgery teams upon transfer to our hospital, surgical intervention was deferred in favor of serial imaging along with antibiotic management. Steroid therapy was also initiated given the concern for cranial nerve compression related to the inflammation superimposed on his cholesterol granuloma. His retro-orbital pain and diplopia with left lateral gaze improved gradually with treatment, and he ultimately received an eight-week course of antibiotics (ceftriaxone with oral metronidazole) followed by a transition to oral antibiotics alone (amoxicillin-clavulanate) while awaiting repeat imaging and potential surgical intervention. Repeat MRI brain midway through his antibiotic course showed stable size of the cholesterol granuloma, a decrease in dural and leptomeningeal enhancement, and resolution of ICA vasospasm and the middle ear and mastoid inflammation (Figure 6). Audiogram testing revealed no hearing loss.

**Figure 7:**
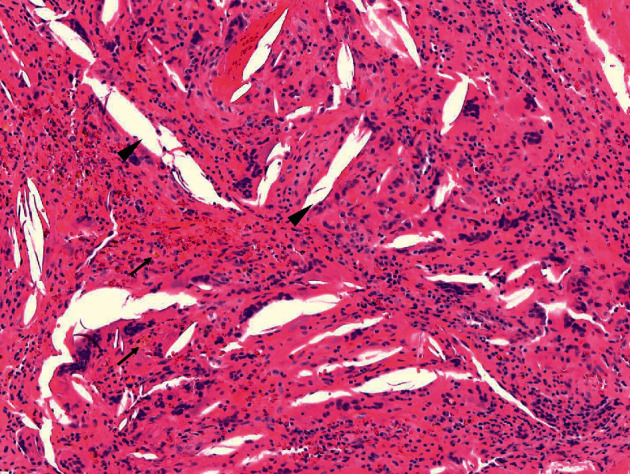
Cholesterol granuloma (100X, hematoxylin and eosin stained), also known as a xanthoma or xanthogranuloma, referring to the cholesterol clefts (arrowheads), lipid-laden macrophages, and multinucleated foreign-body giant cells. In addition, these lesions can demonstrate varying amounts of histiocytes, hemosiderin-laden macrophages (arrows), fibrosis, and calcification. Cholesterol granulomas can be locally destructive, but are typically painless lesions that remain subclinical until discovered incidentally.

Following clinical and radiologic resolution of his meningitis, he ultimately underwent a combined operation performed by neurosurgery and otolaryngology, with endoscopic transsphenoidal drainage of hemosiderin-stained brown motor oil contents from the left petrous apex. Following evacuation of the cyst contents, the left petrous apex defect was marsupialized using a right middle turbinate mucosal graft harvested earlier in the procedure. Tissue collected intraoperatively later demonstrated xanthogranulomatous inflammation consistent with the radiologically suspected cholesterol granuloma (Figure 7).

## 3. Discussion

This patient presented initially with fever, deep retro-orbital pain potentially consistent with a trigeminal neuralgia, as well as eventual ipsilateral abducens palsy. He had no ear pain or discharge preceding these symptoms. Imaging confirmed an expansile PA cholesterol granuloma with smooth, chronic-appearing bone erosion and contiguous dural and leptomeningeal enhancement. His presentation was ultimately consistent with Gradenigo's syndrome occurring concomitantly with *Haemophilus influenzae* meningitis. Despite a lack of clinical ear symptoms or physical exam findings concerning for OM, he was noted to have radiologic findings consistent with possible ipsilateral middle ear infection. The pathogenesis of petrous apicitis has been described by some as the spread of bacterial organisms from the middle ear to PA air cells (pneumatization of the petrous bone occurs in approximately one-third of the population), while others have suggested that vascular (specifically, venous) channels may play a role, as petrous apicitis can rarely occur in those with non-pneumatized petrous apices [4, 19].

PA cholesterol granulomas are rare lesions thought to form as the result of extensive PA pneumatization, which exposes marrow-filled spaces and triggers hemorrhage, obstructing the PA outflow tract and leading to degradation of hemosiderin and cholesterol and a resulting inflammatory granulomatous reaction [10]. Most are slow growing over decades, and smooth bone erosion in large PA cholesterol granulomas is typical. They may remain asymptomatic or present with hearing loss, dizziness or imbalance, tinnitus, headache, facial pain or parasthesia, or diplopia [10].

Following the introduction of the *H. influenzae* serotype B (Hib) vaccine in the early 1990s, the unencapsulated group of nontypeable *H. influenzae* (NTHi) has increasingly been linked to invasive disease over Hib strains [22]. While invasive disease caused by NTHi occurs mostly in the newborn and elderly populations, NTHi nasopharyngeal carriage rates appear to be rising in healthy adults as well [22]. This patient was ultimately found to have an NTHi strain upon further testing of his CSF. Given the patient is an immunocompetent and otherwise healthy adult, we favor that, rather than through hematogenous spread, his meningitis occurred in the setting of colonized middle ear and mastoid air cells communicating with a previously asymptomatic erosive PA cholesterol granuloma, with subsequent direct introduction of bacteria into the meninges and CSF.

This case demonstrates the impact of pre-existing petrous lesions on the risk for development of Gradenigo's syndrome, as well as of potential complications of petrous apicitis including meningitis, even in an era of antibiotics when advanced complications of OM are less common. While large-enough petrous lesions can eventually compress the same traversing nerves and can cause GS symptoms in the absence of recent symptomatic OM, it is reasonable to deduce that bone erosion associated with this patient's cholesterol granuloma made him more susceptible to the introduction of the bacterial organisms from his middle ear and mastoid cavity to his meninges. This patient reported no prior symptoms of a lateral rectus palsy until the superimposed inflammation presumably compounded the impact of this lesion on his abducens nerve.

Surgical drainage for cholesterol granulomas is typically reserved for patients experiencing symptoms of the compressive effects of the lesion on adjacent structures [23]. The largest case series published on 40 patients with petrous apicitis indicates surgery was typically considered only when patients failed antibiotics alone. However, this series excluded patients with cholesterol granulomas [4]. Another review encompassing management of 38 patients with GS was more evenly split between medical management alone and a combined medical and surgical approach [24]. Early recognition of a clinical presentation of GS, classical or otherwise, allows for more prompt radiologic diagnosis of petrous involvement and consideration for additional surgical intervention. Despite the advent of effective antibiotics for typical organisms that colonize the middle ear, for patients with similar expansile petrous lesions contributing to GS, combined medical and surgical management may be necessary to prevent risk of recurrent infectious and mechanical complications.

## Data Availability

All data underlying the results are available within the article, and no additional source data are required.
